# Evaluation of an Electronic Health Record Referral Process to Enhance Participation in Evidence-Based Arthritis Interventions

**DOI:** 10.5888/pcd18.200484

**Published:** 2021-05-13

**Authors:** Lesha E.K. Spencer-Brown, Jenna E. Brophy, Patt E. Panzer, Michael A. Hayes, Jonathan L. Blitstein

**Affiliations:** 1 National Recreation and Park Association, Programs and Partnerships, Ashburn, Virginia; 2 Now with Administration for Community Living, Department of Health and Human Services, Washington, District of Columbia; 3 RTI International, Food, Nutrition and Obesity Policy Research, Research Triangle Park, North Carolina; 4 Patt Panzer Associates, Wilmington, Delaware; 5 RTI International, Public Health Research Division, Research Triangle Park, North Carolina; 6 Now with Insight Policy Research, Arlington, Virginia

## Abstract

**Purpose and Objectives:**

Effective community-based programs to manage arthritis exist, but many adults with arthritis are unaware that these programs are available in their communities. An electronic health record (EHR) referral intervention was designed to strengthen health care and community-based partnerships and increase participation in these arthritis programs. The intervention was developed in response to a national effort that aimed to enhance the health, wellness, and quality of life for people with arthritis by increasing the awareness and availability of, and participation in arthritis-appropriate evidence-based interventions.

**Intervention Approach:**

The National Recreation and Park Association recruited 4 park and recreation agencies and their health care partners to implement an EHR-based retrospective and point-of-care referral intervention. Eligible for referral were adults aged 45 or older with an arthritis condition who were seen by a physician within the past 18 months, and were living within the park and recreation service area. After health care organizations identified eligible adults, they either mailed communication packages describing the availability and benefits of the intervention and conducted phone calls to encourage arthritis-appropriate intervention participation or counseled and referred patients during an office visit.

**Evaluation Methods:**

The pilot was assessed by using semi-structured interviews with key intervention staff members and the Consolidated Framework for Implementation Research.

**Results:**

Our approach resulted in referrals for 3,660 people, 1,063 (29%) of whom participated in an intervention. Analysis of key informant interviews also highlighted the specific contextual factors, facilitators, and barriers that influenced the adaptation and overall implementation of the referral intervention.

**Implications for Public Health:**

Our pilot demonstrates that successful coordination between health care organizations and community-based organizations can promote awareness of and participation in community-based programs. An understanding of the contextual factors and lessons learned can be used to inform processes that can lead to more effective and sustainable health care and community-based partnerships.

**Summary**

**What is already known about the topic?**

Use of electronic health record referrals is an effective way to increase awareness of and participation in evidence-based programs offered in community settings.

**What is added by this report?**

Our evaluation adds to the body of knowledge about contextual factors that affect the implementation and effectiveness in sustainability of referral processes and health care and community-based partnerships.

**What are the implications for public health practice?**

Although referral interventions increase awareness of and participation in community-based programs, the context in which referrals are implemented greatly influences outcomes, and contextual factors should be an integral part of the planning and execution processes.

## Introduction

Arthritis is a leading cause of disability that affects nearly 23% of the US adult population. Arthritis is associated with a lower quality of life resulting from pain, stiffness, swelling of the joints, and limitations in everyday activities. People with arthritis are more likely to experience an injury related to a fall and report co-occurring chronic conditions like diabetes or obesity. In 2013, arthritis-attributable medical costs and arthritis-attributable lost wages totaled $304 billion (1).

The Centers for Disease Control and Prevention (CDC) recommends that people with arthritis engage in joint-friendly physical activity such as evidence-based physical activity and self-management education programs referred to as “arthritis-appropriate evidence-based interventions” or AAEBIs (2). Evaluation of AAEBIs, such as Walk With Ease, Active Living Every Day, Fit & Strong! and EnhanceFitness in community-based settings have shown that participation increases self-efficacy, strength, balance, and physical ability and reduces disability, pain, and fatigue associated with arthritis (3–6). Despite the benefits, participation in AAEBIs remains low, with only 1 in 10 adults with arthritis taking part in these programs (7). Recent research, however, has indicated that adults with arthritis are more likely to become physically active when recommended to do so by a health care provider (8–10).

Health care organizations and community-based organizations share the goal of improving the health of the communities they serve. Community-based organizations play a vital role in community health by creating a continuum of care where people live, work, play, and worship. They allow community members to engage in programs, like AAEBIs, that might improve their health (11–15). Over the last decade, efforts to strengthen partnerships between health care organizations and community-based organizations have increased because of the increase in value-based health care and the demonstrated evidence of improved coordination of and access to health and well-being programs and services, like AAEBIs (16–19). In 2018, researchers studying the effects of a partnership between Baylor Scott & White Health, and Dallas Parks and Recreation found that integration of primary care in a recreation center resulted in a reduction in emergency department use and inpatient care among patients who used the services (20). Research has shown that the use of an electronic referral pathway is a viable and sustainable way to strengthen health care organization and community-based organization partnerships and increase the awareness of and referral into critical community-based chronic disease programs (21–23). Studies have also found that referrals by health care organizations are among the most successful strategies for identifying and enrolling people into evidence-based chronic disease programs such as the National Diabetes Prevention Program (24,25).

CDC funds both national- and state-level organizations to increase awareness and availability of AAEBIs and engage health care providers to increase physical activity counseling and referrals to AAEBIs (26). The NRPA is a CDC-funded national partner that supports the dissemination of AAEBIs through local parks and recreation organizations and their partners. In 2018, NRPA began fostering referral partnerships among health care organizations and community-based organizations (specifically local park and recreation agencies) by developing a referral intervention that used EHRs to identify individuals with specific forms of arthritis to be referred to 1 or more of the AAEBIs. The referral intervention was tested by 4 pilot sites, 1 community-based park and recreation organization, and more than 1 health care organization, located in the West, Midwest, South, and Southwest regions of the United States. The sites were selected on the basis of existing community-based health care organization relationships, experience implementing AAEBIs, staff capacity to expand the AAEBI offerings, and interest and readiness of the health care organization to establish or expand referrals to the park and recreation agencies. Implementation included 2 cohorts: cohort 1 occurred April 2018 through December 2018, and cohort 2 occurred March 2019 through December 2019.

Evaluation of the referral intervention varied in each cohort but occurred mostly after health care organizations initiated referrals to the AAEBIs and participation in the programs began. The pilot sites assisted NRPA, the lead agency; Patt Panzer Associates, the health care consultant; and RTI International in conceptualizing and implementing the evaluation of the referral intervention. Our study examined the implementation of the EHR-based referral intervention through the lens of the Consolidated Framework for Implementation Research (CFIR), a conceptual framework developed to guide systematic assessment of multilevel implementation constructs (27,28).

## Purpose and Objectives

The purpose of our pilot was to establish and demonstrate the potential for community-level partnerships between health care organizations and community-based organizations that promote engagement in AAEBIs among older adults with arthritis by implementing a referral intervention. NRPA, and Patt Panzer Associates established the pilot’s goal and a set of objectives based on the goals of NRPA. The objectives were 1) to develop and implement a referral intervention, 2) to examine the implementation and effectiveness of the intervention, and 3) to examine the facilitators and barriers to implementation and sustainability.

CFIR identifies factors that might influence intervention implementation and effectiveness. CFIR is often used to guide formative evaluation, assess the extent to which implementation is effective in a specific context, and build the implementation knowledge base across multiple studies and settings. CFIR has been used in implementation evaluation studies of health care research to translate results into meaningful patient care outcomes (29–31). The evaluation discussed in this article focused on specific constructs of several CFIR domains: intervention characteristics, inner setting, outer setting, and process of implementation (Table). We applied a modified approach of CFIR and its intervention approach, evaluation methods, and results.

**Table:** Consolidated Framework for Implementation Research^a^ to Enhance Participation in Evidence-Based Arthritis Interventions, April 2018–December 2018 and March 2019–December 2019

Construct	Description
**Intervention characteristics**	
Intervention source	Key stakeholders perceptions about whether the intervention is developed externally or internally
Evidence strength and quality	Stakeholder perceptions of the quality and validity of evidence supporting intervention will have desired outcomes
Relative advantage	Stakeholder perception of the advantage of implementing the intervention rather than an alternative solution
Adaptability	Degree to which an intervention can be adapted, tailored, refined, or reinvented to meet local needs
Trialability	Ability to test the intervention on a small scale within the organization, and be able to reverse course (undo implementation) if warranted
Complexity	Perceived difficulty of implementation, reflected by duration, scope, radicalness, disruptiveness, centrality, intricacy, and number of steps required to implement
Cost	Costs of the intervention and costs associated with implementing the intervention including investment, supply, and opportunity costs
**Outer setting**	
Patient needs and resources	Extent to which patient needs are accurately assessed and prioritized by the organization, and how barriers and facilitators are assessed to help or hinder meeting those needs.
Cosmopolitanism	Degree to which an organization is networked with other external organizations
**Inner setting**	
Structural characteristics	Social architecture, age, maturity, and size of an organization
Networks and communications	Nature and quality of webs of social networks and the nature and quality of formal and informal communications within an organization
Readiness for implementation	Tangible and immediate indicators of organizational commitment to its decision to implement an intervention
**Process**	
Engaging	Attracting and involving appropriate people in the implementation and use of the intervention through a combined strategy of social marketing, education, role modeling, training, and other similar activities
Reflecting and Evaluating	Quantitative and qualitative feedback about the progress and quality of implementation accompanied by regular personal and team debriefing about progress and experience

a Adapted with permission from Damschroder et al (27). Fostering implementation of health services research findings into practice: a consolidated framework for advancing implementation science.

## Intervention Approach

We characterized the referral intervention approach using several CFIR intervention constructs: source, evidence of strength and quality, relative advantage of implementing the intervention versus an alternative, adaptability, trialability, complexity, and cost (27,28). The intervention was designed to promote the awareness of and participation in AAEBIs at park and recreation sites by engaging health care organizations and their patients. The intervention was developed in consultation with the pilot sites that wanted to implement a simple, adaptable referral process that would make use of EHR capabilities while also targeting a substantial number of people with specific conditions who would benefit from the AAEBIs. Although many of the health care organizations involved routinely used their EHRs to identify and refer patients to various in-house health programs and services including diabetes programs, immunizations, and mammograms, none had ever used the EHR to identify a large number of patients and subsequently refer them to a community-based chronic disease program. Additionally, all the park and recreation agencies had experience implementing AAEBIs or other evidence-based programs. The AAEBIs have been rigorously evaluated in clinical and community-based settings and are supported by public health entities, including CDC, which adds to the quality, strength, and advantage of the intervention in producing the desired results for people with arthritis.

The referral intervention was intended to be highly adaptable and of minimal complexity. It comprised 4 phases: 1) referral intervention planning, 2) query development, 3) connection to community-based program, and 4) program engagement and feedback. The referral intervention was developed for community-based organizations and health care organizations to allow flexibility where the health care organization EHR was not customized to refer to AAEBIs or other community-based chronic disease programs; no existing business agreement was in place to allow the transmission of protected health information from the health care organization to the community-based organization; the community-based organization did not have a streamlined and Health Insurance Portability and Accountability Act of 1996 (HIPPA)-compliant process for receiving referrals: or the health care organization could use its staff (eg, health coaches, patient care navigators, community health workers or other available staff) to navigate patient referrals (Box).

**Box. Summary of Referral Intervention Planning**

Health care organizations (HCOs) and community-based organizations provide detailed information for referral intervention steps (establishing referral criteria, creating a timeline and workﬂow; identifying staff roles, educational opportunities, locations of program offereings; identifying measures of success; establishing a partner communication plan and feedback communication; and developing communication materials).

**Query Development**

HCO staff (eg, electronic health record [EHR] specialists, clinical managers) use referral criteria identiﬁed in the planning phase to generate a report of eligible patients. Criteria follow:

- **Arthritis diagnosis.** Major forms of arthritis should be documented by ICD-10 codes. Some major forms of arthritis are rheumatoid arthritis, osteoarthritis, and gout.
- **Zip code.** Park and recreation service areas are matched to patients identiﬁed to ensure residence is in an area serviced by the agency.
- **Age.** In the pilot, patients 45 years or older were identiﬁed as meeting criteria.
- **Last seen with condition.** The date the identified patient was last seen by a health care provider.

**Connection to Community-Based Program**

Identiﬁed patients are informed of arthritis appropriate evidence-based interventions (AAEBIs) in 2 ways.

- **Retrospective referral.** HCO staff (eg, health coaches, community health workers, patient care navigators) uses EHR query report to mail communication packages consisting of AAEBI informational flyer and personalized letter; HCO follow up with referred patients by phone or other channels of communication (eg, patient portal) to encourage participation.
- **Point-of-care referral**. HCO staff (health coaches, medical assistants) generates weekly report that identiﬁes patients due for an in-person office visit; health care providers counsel those patients about the AAEBIs and refer them during the visit.

**Program Engagement and Feedback**

- Referred patients contact the park and recreation agency to enroll in an AAEBI.
- Park and recreation agency sends aggregate data to referring HCO after AAEBI completion.

Because this intervention was intended to be adaptable, sites were able to tailor different aspects such as patient criteria (eg, age, timeframe) and referral type (retrospective vs point-of-care), depending on their resources, preferences, and knowledge of their patient population. As it relates to trialability, the intervention was implemented as a pilot that included iterations of phases 2, 3, and 4 so sites could use lessons learned to improve and promote successful adaptation. Additionally, the intervention was designed to be relatively straightforward to implement, without requiring extensive training or changes to the organizations’ workflows. Costs to implement the referral intervention were limited to intervention materials and staff time. For park and recreation agencies, costs included AAEBI trainings; program materials such as manuals and equipment, marketing materials, and in-kind staff time; and minimal staff compensation as needed for expanded capacity to facilitate the AAEBIs. For health care organizations, costs included in-kind staff time to manage EHR queries and referral outreach either by telephone or by mail and material costs for any health care organization marketing, such as pamphlets. Examples of health care organization staff members primarily involved in the planning and implementation of the referral intervention were clinical managers, operations managers, EHR data specialists, health coaches and patient navigators, volunteers, behavioral health and health education faculty, and care managers.

## Evaluation Methods

From July through August of 2018 (Cohort 1) and from August through October 2019 (Cohort 2), qualitative evaluation methods were used to capture the experiences of the staff of all the park and recreation agencies and health care organizations involved in implementation; no sites were excluded. The evaluation assessed program planning and implementation, participant recruitment, internal and external collaboration and communication, facilitators, barriers, successes, challenges to implementation, sustainability, and recommendations for scaling. Semistructured interview guides consisted of open-ended questions created for each of the 7 role types: park and recreation manager, AAEBI instructor, and the following roles from health care organizations: physician, manager, data specialist, manager and data specialist hybrid role, and health coach or volunteer. Pilot leads from the sites identified staff members who could serve as knowledgeable key informants based on their role in the implementation process. Across the 2 cohorts, 25 key informants were identified. Two moderators conducted 23 interviews with the staff at the 4 pilot sites for a participation rate of 92%; each interview lasted between 15 and 75 minutes. The interviews were conducted shortly after the identified patients had been referred and AAEBI participation had begun. RTI’s institutional review board approved the study.

Evaluation data consisted of interview notes and transcripts. The information was reviewed and transferred into a data abstraction matrix created by using Microsoft Excel (Microsoft Corporation). The Excel matrix contained a form for each participant type, questions from the corresponding interview guide in the row headers, and a column for each participant. Additionally, information learned from monthly check-in calls and email updates was included. Data synthesis focused on interviewee responses to questions and follow-up probes. In summarizing the findings, the content in terms of themes, frequency, and types of causal and logical statements interviewees expressed were considered, noting any regularities, patterns, explanations, and propositions contained in the data. When interviewee responses to 1 question helped address another, answers across the different questions were integrated.

## Results

During the implementation period, a total of 3,660 people were referred from health care organizations to an AAEBI that was offered at a park and recreation agency. Of those referred, 1,063 participated in an AAEBI, resulting in a referral engagement rate of 29%. Sites reported that reasons for AAEBI interest and engagement from the referred participants varied but were largely a result of 1) increased awareness of the availability of AAEBIs and knowledge about their benefits, 2) improved credibility for the AAEBIs arising from referrals from health care providers, specifically for arthritis, 3) a strong sense of community cohesion and trust for the park and recreation agency, 4) adaption of the referral intervention based on understanding of the target population, and 5) reduction in barriers (eg, cost, transportation) to participation in the AAEBI.

Overall, staff members from pilot sites were optimistic about referral interventions, and many believed they were effective. According to 1 health care organization manager, “To get people into workshops is a very challenging thing to do. The EHR is a critical piece, but it is . . . all of it, the flyers, the calls, the letters. We say it is effective.” Similarly, a park and recreation manager stated, “I think it has taken all the programs to another level. It opened an opportunity to reach out to more patients that we previously did not have. It is overwhelming in a good way. The people are responding. They are calling. They are participating. They are excited. It’s just been unbelievable to see how it just kind of played out.”

Furthermore, sites mentioned their hopes to expand or create similar interventions for populations for whom increased physical activity and self-management education could improve health outcomes (eg, diabetes). Both the health care organizations and park and recreation agencies were aware of their limitations in community outreach and understood the value of being engaged in their communities. As a health care organization manager noted, “Our mission is building healthier communities together and that is not something we can go at alone. I feel like this is the future, partnering to allow everyone to do what they are best at. We don’t have to be the best at everything.”

Constructs from CFIR’s intervention characteristics domain (evidence strength and quality, relative advantage, adaptability, trialability, complexity, cost) reflect the perceptions of quality, flexibility, and potential benefits of the intervention. Among the sites, all park and recreation managers and nearly all health care organization managers indicated that the referral process was not difficult to implement. The sites also indicated that the pilot aligned with their mission and goals, and the intervention was a good opportunity to form a partnership that could address a gap in services for patients with arthritis and other chronic conditions. Health care organization staff also noted that an advantage of this intervention was the fact that the programs being referred to were evidence-based programs vetted and supported by public health entities including the CDC, and partnerships were still feasible despite their limited capacity to implement streamlined EHR referrals to the park and recreation agencies.

In terms of adaptability, each site tailored the intervention to best fit the needs of their organization’s workflow and patient population. For example, a health care organization manager believed their patient population would not be receptive to the use of the retrospective EHR referral; instead, they used a modified version of the point-of-care referral, in which individuals were counseled and referred during the clinic visits.

Pilot sites reported that the trialability of the intervention was appealing because it was easily reversible, and they could leverage preexisting resources needed for implementation. Many of the organizations also planned for a small and scalable approach to implementing the intervention. For example, some of the health care organizations limited implementation to either 1 clinic (eg, the rheumatology clinic) or to specific providers within a practice. All of the park and recreation agencies limited the AAEBI offerings to select locations to allow for time to reflect on and promote successful adaptation of the intervention.

At 1 site, the health care organization manager and park and recreation manager summarized these constructs well: “I think it’s a fairly low cost and easy process for us to make those referrals. And again, it’s good for our patients,” and “You know we’re all on a shoestring budget at times and we may have to modify some things, but we’re going to continue to try to do our best to keep these programs.”

An aspect of the referral intervention that acted as a barrier to implementation was the inability of the park and recreation agency to receive referral information directly, which in turn affected their ability to follow up and encourage participation in an AAEBI. The health care organizations had dedicated staff members who were able to follow up with participants, but many interviewees thought that the knowledge and expertise of the park and recreation agency as the AAEBI provider could have encouraged people who did not initiate contact to learn more about the AAEBIs. To address this barrier, many health care organizations and park and recreation staff members recommended establishing formal partnerships (eg, business associate agreements) between the organizations to allow for HIPPA–compliant transmission of the referral information.

Two constructs in CFIR’s outer setting domain influenced the implementation of the intervention: 1) patient needs and resources and 2) cosmopolitanism. Some considerations made by pilot sites included ensuring that patients had choices for AAEBI engagement, cost of the AAEBIs was minimal, trust was maintained, and access was prioritized. For example, some of the park and recreation agencies offered the AAEBIs at no cost to participants to remove cost as a barrier for participation. In addition, the query only considered the zip codes of people who lived in an area serviced by the park and recreation agency so that people referred would be able to access the AAEBI location. The park and recreation agencies also offered 1 or more of the AAEBIs so that those referred could choose the AAEBI that best suited their physical needs and schedules.

Another important factor that sites considered was trust. To garner trust, most sites personalized the letters sent to referred people and included information about the research demonstrating that AAEBIs help people manage their arthritis symptoms. Health care organizations also acknowledged their willingness to follow up with referred people, learn about their experiences, and if necessary, help them find a program that could work for them.

Sites recognized the barriers with patient needs and resources such as transportation and the person’s readiness to change. To address barriers, sites presented information in the program flyer about resources available for transportation, and they learned about patient readiness to change during the follow-up telephone calls or in-office counseling.

All sites had strong external networks with organizations that aided implementation. For example, 1 park and recreation agency collaborated with the health care organization’s regional health education hub that served as a centralized coordination center for referrals to health education resources offered by community-based organizations throughout several counties. Through the hub, the health care organization was already involved in identifying and referring people to community-based organizations to address various social determinants of health. Describing the hub’s importance, 1 health care organization manager stated, “It allowed us to very easily pick up this . . . program. I don’t know what we would have done without it. Having a hub and a health system to lead programming of community-based organizations, instead of having to go to each individual clinic is efficient.*”* Other sites were also previously involved in external networks, such as a health action board or additional initiatives that supported community health improvement efforts. These external networks allowed the sites to implement the intervention more easily, either because of the existing structures or the knowledge from previous efforts or alliances. 

Our findings aligned with the following constructs of CFIR’s inner setting domain: structural characteristics of the network and communications and readiness for implementation. Sites involved in implementation varied in structural characteristics. Health care organizations had populations ranging from 5,000 to more than 20,000 patients. The health care organizations ranged from small, privately owned practices with fewer than 20 health care staff members in single locations, to large hospital systems with more than 200 beds in multiple locations, including specialty clinics and outpatient facilities. Additionally, many of the health care organizations were involved in community-level initiatives to address chronic diseases among their patient populations. They employed health coaches, patient care navigators, health education coordinators, and people in similar roles, all tasked with connecting the referred patients to the AAEBIs. The park and recreation agencies involved also varied, from staff roles to serving varying population sizes and zip codes. Some agencies also had fewer than 5 community centers, and others had more than 10 community centers.

Three of the 4 community-based organizations had worked with their health care organization in the past through formal partnerships, but all 4 stated that the intervention helped advance those partnerships. One park and recreation manager who already had a formal partnership stated, “We did not know they had a rheumatology department . . . So, this pilot has helped us have a direct pipeline to those clients.”

Most managers noted a high degree of communication between organizations with frequent phone calls, email communication, and in-person visits. Another park and recreation manager noted that a strong advocate and champion in both the health care organization and the park and recreation agency was needed to make the intervention function well. 

Regarding readiness for implementation, findings aligned with the following themes: leadership engagement, available resources, and access to knowledge and information. All sites had leadership that supported the effort, and some leaders were directly engaged in program implementation. For example, a health care organization manager explained, “Usually the leadership here, the 2 owners, another manager, and myself, we get to help devise strategy at the clinic. I get to be a part of the process of selection of what programs we want to bring into the clinic.” 

The decision to implement the intervention was also influenced by the ease of access to information and knowledge about how to incorporate the intervention into workflows. Another factor that aided commitments was the availability of resources, health care organization staff education, experienced and appropriate health care organization and park and recreation staff, and documentation of the referral intervention and AAEBIs. 

Two constructs in CFIR’s process domain influenced the implementation of the intervention: engaging and reflecting and then, evaluating. Attracting and engaging the appropriate people in the referral intervention significantly influenced implementation and success. All sites reported having a champion(s) who secured buy-in from leadership and staff members who would play a role in the intervention and spearhead the implementation process. For the health care organizations, these people were strong proponents for community integrated health strategies whose roles included population health-level responsibilities. Champions engaged members of their teams who helped them carry the referral intervention forward. Additionally, park and recreation managers expressed a high level of commitment and strongly believed that engaging with health care organizations was rewarding and furthered their community’s health improvement goals. As 1 park and recreation manager reported, “It reinforces the idea that this is health care — that wellness is health care.”

Because this was a pilot intervention, feedback about the progress and quality of the referral intervention was frequently discussed during the monthly project check-ins with NRPA and among the sites themselves outside of their monthly meetings. One process improvement resulting from feedback was that after reviewing the query-generated report, some health care organizations used their intimate knowledge of the patients to remove those who initially appeared to be a good fit for the AAEBIs but were deemed inappropriate by the clinical reviewers, for various reasons, including being home-bound or having other health conditions or events that would limit active participation (eg, recent surgery).

Reflection and evaluation also led to recommendations for future implementation of the referral intervention as resources allowed, including 1) leveraging EHR prompts and drop-down menus for referrals or other ways to streamline referrals (eg, URL web links, coordination platforms like Unite Us), 2) identifying and investing in staff members (eg, community health workers, health education specialists, health coaches) who could prioritize community-based resources and connect with patients, 3) using a multifaceted approach to reinforcing a referral (eg, using waiting room TVs, informational flyers in high-trafficked areas, patient portals, exam room rack cards, websites, and news outlets), 4) identifying criteria that would allow for the consideration of social determinants of health in patient identification, and 5) collecting data to help build the case for a continued referral intervention that was relevant to all partners.

## Implications for Public Health

Effective and sustainable health care organization and community-based organization partnerships that address the drivers of health and health behaviors are critical if we are to improve the health of our communities. Establishing a referral intervention is 1 way in which organizations with aligned missions and visions can work together to achieve their goals. The relative advantage of leveraging the EHR system is increased access to and ease in connecting large numbers of people to community-based resources in collaboration with their health care providers as opposed to more burdensome and less sustainable referral pathways that continue a cycle in which people who need community supports the most remain uninformed and without access. The findings of this evaluation demonstrate that the referral intervention was effective in promoting participation in the AAEBIs. Although this intervention did not use the full capabilities of the EHR, it does provide an achievable and effective referral process for many partnerships that may not have the resources to use the full capabilities of the EHR.

Furthermore, this evaluation found that the context in which referral interventions are implemented influences their outcome and sustainability. These contextual factors drive change and can either support or restrict health care organizations and community-based organizations in their efforts to foster referral partnerships that enhance services to their communities. Specifically, the intervention is crucial to adaptation; its supporting evidence and advantage must be of high quality and it must be highly adaptable and simple in design. The intervention must solve high-priority needs, be supported by the community and the organization from the top down, and must take into consideration the unique needs of the intended audience. Organizations themselves must be well positioned to adapt and sustain the intervention.

Finally, this evaluation highlights the need for 1) expanded investments in staff members (ie, community health workers, health education specialists, health coaches) who are able to prioritize patient outreach and connection to community-based resources, 2) research to enhance understanding of the factors that can influence patient readiness to change and subsequent participation in community-based programs, and 3) multisector collaborative efforts by health care organizations, community-based organizations, EHR software developers, vendors, and others to maximize use of EHR systems and data elements to identify patients who are most appropriate for referrals and to streamline community-based organization and health care organization referrals.

Our work had several limitations. First, the pilot was not initially evaluated by using CFIR. CFIR was later applied to better categorize the contextual factors that affected the referral implementation. Second, demographic information for participating patients was unavailable because of inconsistent data collection procedures. Third, like all implementation research, the findings reflect the people, places, and organizations involved in the intervention. Although people, places, and organizations might vary greatly, organizations have many potential commonalities, and results may be useful to other organizations interested in applying lessons to their own projects. Lastly, at some sites, implementation was much slower than initially expected, and evaluation activities overlapped with progress toward pre-identified goals. At times, pilot sites were asked questions about activities that were underway or had not yet occurred. Almost all of the sites reported that this pilot was the beginning of a process that would be built on and continued over time, similar to other behavioral interventions.
